# Liquid biopsy: current technology and clinical applications

**DOI:** 10.1186/s13045-022-01351-y

**Published:** 2022-09-12

**Authors:** Mina Nikanjam, Shumei Kato, Razelle Kurzrock

**Affiliations:** 1 Division of Hematology-Oncology, University of California San Diego, La Jolla, 1200 Garden View Road, Encinitas, CA 92024 USA; 2 Medical College of Wisconsin Cancer Center, Milwaukee, WI USA; 3 WIN Consortium, Paris, France

**Keywords:** Liquid biopsy, CTC, cfDNA, ctDNA, Precision medicine

## Abstract

Liquid biopsies are increasingly used for cancer molecular profiling that enables a precision oncology approach. Circulating extracellular nucleic acids (cell-free DNA; cfDNA), circulating tumor DNA (ctDNA), and circulating tumor cells (CTCs) can be isolated from the blood and other body fluids. This review will focus on current technologies and clinical applications for liquid biopsies. ctDNA/cfDNA has been isolated and analyzed using many techniques, e.g., droplet digital polymerase chain reaction, beads, emulsion, amplification, and magnetics (BEAMing), tagged-amplicon deep sequencing (TAm-Seq), cancer personalized profiling by deep sequencing (CAPP-Seq), whole genome bisulfite sequencing (WGBS-Seq), whole exome sequencing (WES), and whole genome sequencing (WGS). CTCs have been isolated using biomarker-based cell capture, and positive or negative enrichment based on biophysical and other properties. ctDNA/cfDNA and CTCs are being exploited in a variety of clinical applications: differentiating unique immune checkpoint blockade response patterns using serial samples; predicting immune checkpoint blockade response based on baseline liquid biopsy characteristics; predicting response and resistance to targeted therapy and chemotherapy as well as immunotherapy, including CAR-T cells, based on serial sampling; assessing shed DNA from multiple metastatic sites; assessing potentially actionable alterations; analyzing prognosis and tumor burden, including after surgery; interrogating difficult-to biopsy tumors; and detecting cancer at early stages. The latter can be limited by the small amounts of tumor-derived components shed into the circulation; furthermore, cfDNA assessment in all cancers can be confounded by clonal hematopoeisis of indeterminate potential, especially in the elderly. CTCs can be technically more difficult to isolate that cfDNA, but permit functional assays, as well as evaluation of CTC-derived DNA, RNA and proteins, including single-cell analysis. Blood biopsies are less invasive than tissue biopsies and hence amenable to serial collection, which can provide critical molecular information in real time. In conclusion, liquid biopsy is a powerful tool, and remarkable advances in this technology have impacted multiple aspects of precision oncology, from early diagnosis to management of refractory metastatic disease. Future research may focus on fluids beyond blood, such as ascites, effusions, urine, and cerebrospinal fluid, as well as methylation patterns and elements such as exosomes.

## Background

**Fig. 1:**
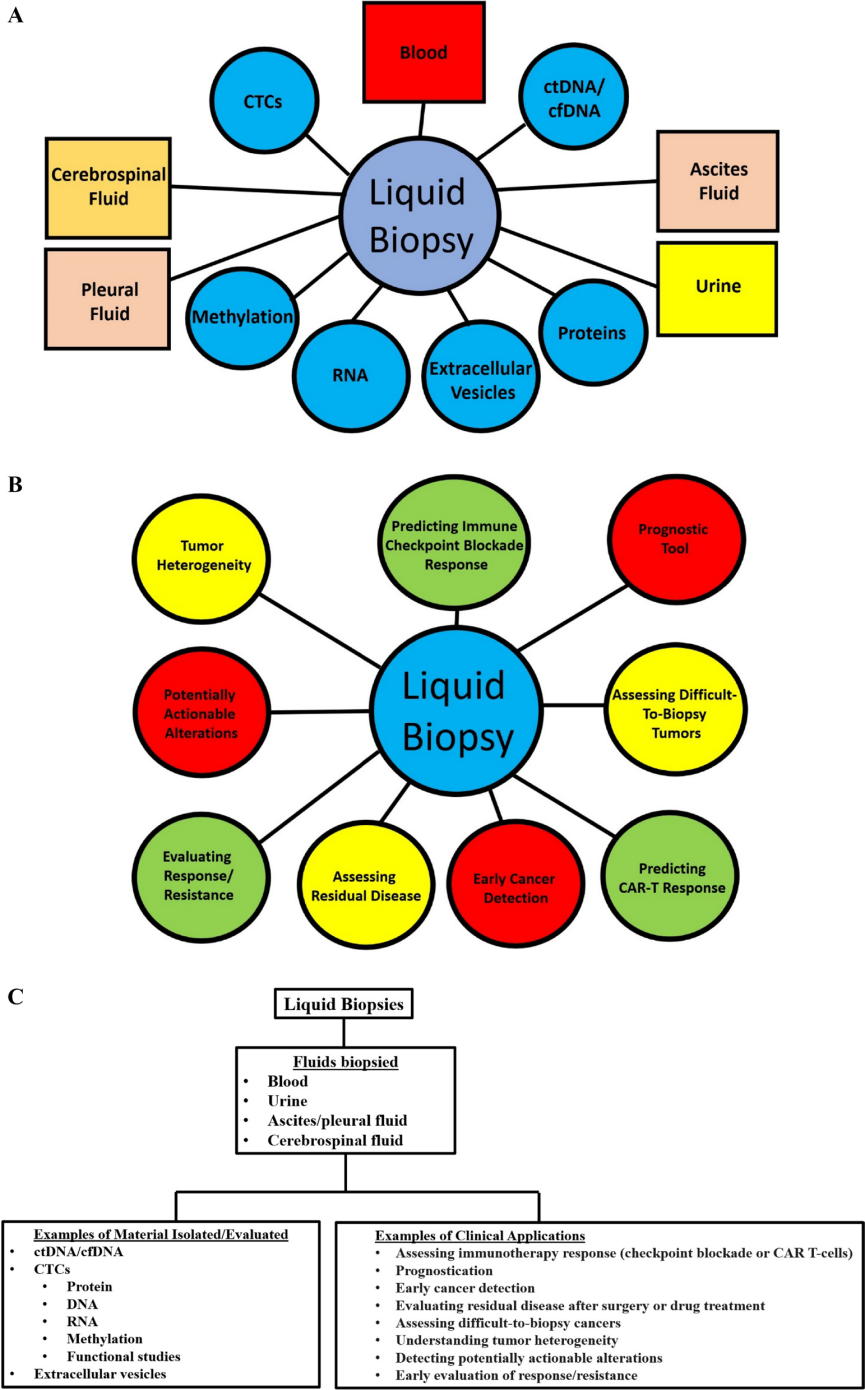
**A** Types of liquid biopsies. Liquid biopsy is most commonly obtained via blood sampling, but can also be derived from urine, CSF, ascites fluid, and pleural fluid. cfDNA/ctDNA, CTCs, RNA, and extracellular vesicles can be isolated from these fluids. Protein expression and methylation patterns can also be assessed with liquid biopsy. Boxes represent examples of fluids biopsied and circles represent examples of materials isolated/evaluated. **B** Clinical applications of liquid biopsies. Liquid biopsies (cfDNA/ctDNA and CTCs) have been utilized for a variety of purposes as noted. **C** Examples of types of liquid biopsies, material isolated/analyzed, and clinical applications. Liquid biopsy is most commonly obtained via blood sampling, but can also be derived from a variety of other fluids. cfDNA/ctDNA and CTCs are the most commonly isolated and analyzed materials. cfDNA: cell-free DNA, ctDNA: circulating tumor DNA, CTCs: circulating tumor cells

Liquid “biopsy” technology, which enables the molecular interrogation of liquid samples (usually blood), has advanced at breathtaking speed, facilitating its routine clinical use in patients with cancer, and rapidly expanding research capabilities that are uncovering the basis of malignant growth. Liquid biopsies are minimally invasive and provide a methodology for obtaining tumor-derived information from body fluids. There are many body fluids that can be biopsied; however, the most commonly used fluid is blood (Fig. 1A, C).

Circulating extracellular nucleic acids (cell-free DNA; cfDNA) and circulating tumor DNA (ctDNA) can be isolated from the blood. cfDNA is DNA freely circulating in the blood, which may or may not be of tumor origin, whereas ctDNA is of tumor origin. Circulating tumor cells (CTCs) can also be isolated from the blood. These are cells that are shed into the blood from tumors and usually only last for 1–2.5 h in the circulation prior to destruction by the immune system, but a small fraction can survive and seed distant metastatic sites. ctDNA/cfDNA and CTCs can be assayed using next-generation sequencing (NGS) [1, 2]. Protein and RNA CTC content can also be assessed, as well as functional CTC characteristics, which can guide the use of potential therapeutic compounds. Differences between ctDNA/cfDNA and CTCs are shown in Table 1 [3, 4].

**Table 1:** Comparison of ctNDA/cfDNA and CTCs [1–4]

	ctDNA/cfDNA	CTCs
Ease of collection/isolation	Easier isolation	More difficult to isolate
Ability to culture	Cannot be cultured	Can be cultured
Predicting therapeutic response	Changes in levels predict response/resistance/relapse	Changes in levels can predict response/resistance/relapse
Ability to assess genomic/transcriptomic/protein data	Can analyze DNA	Can analyze DNA, RNA, and protein
Ability to assess functional data	No	Yes
Ability to assess methylation	Yes	Yes
Ability to perform fluorescent in situ hybridization analysis	No	Yes
Ability to perform single cell analysis	No	Yes
Ability to perform chromosomal analysis	No	Yes
Challenges in collection/interpretation	Cell death under therapy can modify ctDNA levels Small quantities of ctDNA in circulation Can be confounded by CHIP	Heterogeneity in CTCs can affect analysis Sampling bias of captured cells (high affinity and larger size)

*CHIP* clonal hematopoiesis of indeterminate potential, *cfDNA* cell-free DNA, *ctDNA* circulating tumor DNA, *CTC* circulating tumor cells

This review provides a summary of current technologies for detecting ctDNA/cfDNA and CTCs from liquid biopsies, along with clinical applications and future directions.

### Technologies for ctDNA/cfDNA detection

The following techniques are among those that have been deployed to evaluate ctDNA/cfDNA: droplet digital polymerase chain reaction (ddPCR), beads, emulsion, amplification, and magnetics (BEAMing), tagged-amplicon deep sequencing (TAm-Seq), cancer personalized profiling by deep sequencing (CAPP-Seq), whole genome bisulfite sequencing (WGBS-Seq), whole exome sequencing (WES), and whole genome sequencing (WGS).

ddPCR can detect as low as 0.01–1.0% of genomic material and is useful for identifying potentially rare mutations and calculating copy number variants [5, 6]. However, it can only be used to evaluate the presence of characterized sequences.

Similarly BEAMing is a relatively sensitive and inexpensive method of screening for known mutations. It combines PCR with flow cytometry and can detect alterations at levels as low as 0.01% with excellent concordance to tissue testing [6] [38, 43].

CAPP-Seq identifies alterations in ctDNA/cfDNA using large genomic libraries and individual patient sample sequence signatures. It statistically assesses well-characterized tumor alterations with DNA oligonucleotides to find patient-specific alterations. It can identify multiple mutations in patients with the same type of cancer and assess tumor heterogeneity. It was previously shown to be capable of identifying tumor burdens prior to medical imaging. It can identify many major mutation types including insertions, deletions, single nucleotide variants, copy variants, and rearrangements, but cannot identify fusions [6, 7].

Tam-Seq allows for a very specific and sensitive analysis (~ 97%) and can detect DNA levels as low as 2% by using primers to tag and identify genomic sequences. It has a high sequencing flux, reduced sequencing time and cost, and can simultaneously sequence millions of DNA molecules. However, the desired sequence needs to be previously characterized for the methodology to work [6, 8].

Whole exome sequencing provides characterization and analysis of all present tumor mutations, thus can identify potential oncogenes and tumor suppressor genes. However, its sensitivity may be lower than other methods since it includes exomic alterations. It is characterized by low cost and high yield [6, 9].

Whole genome sequencing evaluates the entire tumor genome to determine characterized and deleterious alterations along with variants of unknown significance. It has great potential for a comprehensive evaluation of all tumor mutations, but is limited by quality assurance, ethical issues, time, and cost. Interpretation of results can be difficult outside of specialized centers[9, 10].

WGBS-Seq is the gold standard in DNA methylation analysis. It provides a single cytosine measurement and has very high accuracy. While it can discover partially methylated domains in cancer cells, DNA may exist in varying degrees of degradation and thus this method can have reduced sensitivity [11, 12].

### Technologies for CTC detection

The following methods have been used to isolate CTCs: immunogenicity, positive enrichment, negative enrichment, enrichment based on biophysical properties (i.e., size, density) [13].

CTC enrichment by immunogenicity is one of the most widely used techniques for isolating CTCs. The cells are captured using specific biomarkers expressed on the cell surface and secure to a device surface or magnetic substance. However, there is no single, universal CTC antigen since a variety of surface markers are expressed by CTCs [13].

There are several methods for positive enrichment of CTCs. AdnaTest uses antibody-coated beads specific to the type of cancer and real-time polymerase chain reaction is run to determine expression patterns on the cells [14]. Magnetic-activated cell sorting (MACS) captures cells with magnetic nanoparticles attached to antibodies [14–16]. MagSweeper is an immunomagnetic enrichment technology which uses a robotic ally-controlled magnetic rod and antibody-coated magnetic beads to isolate CTCs [14]. The CellSearch system uses ferrofluid nanoparticles to separate epithelial cell adhesion molecular (EpCAM) cells from other blood cells after centrifugation [10]. Target Selector, CTC platform (Biocept) uses an antibody cocktail to capture CTCs which target EpCAM along with other mesenchymal and stem cell tumor-associated and cell-type-specific markers. It can also assess various biomarkers at the protein and DNA levels within the microfluidic channels [17].

There are two main negative enrichment strategies for isolating CTCs. The EasySep system uses a magnetic technology. It incubates antibodies targeting CD45 cells and magnetic nanoparticles with the samples. The Quadrapole Magnetic Separator functions as a magnetic flow cytometer to detects immunomagnetically labeled cells [14].

CTCs can also be separated based on biophysical properties. CTCs are generally larger than background cells which can allow for centrifugation, microfiltration, and dielectrophoresis techniques [18, 19].

Once isolated, aberrations in the CTCs can be identified with DNA, RNA, or protein techniques. DNA can be amplified and analyzed [20]. Fluorescence in situ hybridization can be used to identify gene amplifications or translocations on CTCs [21]. Transcriptome/RNA profiling can be performed by sequencing or in situ hybridization [22].

CTCs can also be used in functional analyses. A prior study evaluated mechanisms of endocrine resistance and late recurrence in an ER + /HER2- breast cancer patient. CTCs were isolated after progression on endocrine therapy and cultured to make a cell line which was utilized to explore signaling pathways [23]. CTCs have been used to establish cell lines for prostate [24], lung [25], breast [26], and colon cancer [27]. An alternative method is xenografting by injecting CTCs into immunodeficient mice which has been explored in breast [28, 29], prostate [28], and lung cancer [30]. These methodologies provide platforms which may allow for evaluation of therapeutic response and resistance.

## Clinical applications of ctDNA/cfDNA and CTCs

There are a multitude of clinical applications for ctDNA/cfDNA and CTCs (Fig. 1B, C). The following will be discussed in this section: differentiating unique immune checkpoint blockade response patterns, predicting immune checkpoint blockade response, assessing shed DNA from multiple metastatic sites/tumor heterogeneity, assessing potentially actionable alterations, serial sampling for response and early detection of resistance for targeted therapy and chemotherapy, using liquid biopsies as a prognostic tool, evaluating difficult-to biopsy patients, early detection of cancer, and predicting CAR-T cell response.

### Differentiating unique immune checkpoint blockade response patterns using serial liquid biopsies

Immune checkpoint inhibitors are increasingly used in oncology because of their ability to activate the immune system, which can then, at times, eradicate even refractory cancers. ctDNA/cfDNA has been explored as a tool to provide early assessments of immune checkpoint response and as a complement to standard imaging studies. Immunotherapy can have unique response patterns that can confound clinical assessment of the patient’s response. For instance, pseudo-progression is an apparent initial increase in tumor size on imaging despite a clinical response to therapy; pseudo-progression may occur due to immune inflammation, which makes the tumor appear larger on imaging, when in fact it is regressing. Additional tools to differentiate progression from pseudo-progression are important for clinical decision making. Hyper-progression is a phenomenon where immune checkpoint blockade leads to an accelerated rate of growth in tumors [31]. Early predictive markers of patients who are undergoing hyper-progression would help transition patients to more effective therapy prior to awaiting imaging results.

Ricciuti et al. used ctDNA to help predict responses to immunotherapy-based treatment for non-small cell lung cancer [32]. Patients undergoing pembrolizumab ± platinum/pemetrexed therapy had ctDNA evaluated at baseline. The % change in ctDNA at first follow-up and % change in tumor target lesions were significantly correlated. Decreases in ctDNA were associated with significantly higher response rates, longer median progression-free survival, and median overall survival as compared to those with an increase.

Kato et al. prospectively evaluated serial cfDNA for variant allele frequency in a pan-cancer cohort receiving immune checkpoint inhibitors. Low vs. high cfDNA-derived average adjusted changes in variant allele frequency was an independent predictor of clinical benefit rate, progression-free survival and overall survival [33].

Zhang et al. [34] evaluated ctDNA pre-treatment and on-treatment in advanced-stage cancer patients on clinical trials of durvalumab ± tremelimumab. Higher pretreatment variant allele frequencies related to worse overall survival, however, on-treatment reductions in variant allele frequency were associated with longer progression-free and overall survival along with objective response rates. Changes in ctDNA were felt to be more dynamic than radiographic changes and a potential molecular response metric for immunotherapy across cancer types.

Jensen et al. used genome instability number (GIN) which was calculated from low-coverage, genome wide sequencing of cfDNA to evaluate response to immunotherapy [35]. GIN represents the cumulative deviations of all copy number alterations across the genome and is influenced by both the magnitude of the copy number alterations and the level of cfDNA in the plasma. Samples were serially collected for patients receiving immunotherapy treatment. While baseline GIN was not predictive of response to immunotherapy, the pattern of dynamic changes in GIN after treatment predicted response. Patients with response generally had continuously decreasing GIN levels or a spike in GIN followed by at decrease near week 6 of treatment. Decreasing GIN levels were also found to accurately predict pseudo-progression. The study found this technology also pinpointed patients with hyper-progression early (at about three weeks post-therapy) by demonstrating a rapid and sustained increase in blood-derived GIN in the first few weeks of therapy.

Thus, ctDNA/cfDNA can provide early and dynamic assessments of response and progression for immune checkpoint therapy, which can be used with imaging studies to guide treatment. In particular, cfDNA may be valuable in determining if a patient has pseudo-progression or true progression, which can be challenging with imaging studies alone.

### Predicting immune checkpoint blockade response based on baseline blood tumor mutational burden or microsatellite status

Determining which patients will derive benefit from immune checkpoint therapy is important for therapeutic decision making. Tumor mutational burden (TMB) detected by next-generation sequencing of tumor tissue has been shown to correlate with response to immune checkpoint inhibitors [36]. Microsatellite instability high (MSI-H) has also been predictive of response to immune checkpoint inhibitors in advanced cancers [37]. Blood-based tumor mutational burden and MSI status has also been explored to determine which patients will benefit from immune checkpoint therapy. Khagi et al. investigated the association between hypermutated ctDNA and immunotherapy response (54–70 genes analyzed) [38]. High numbers of either total alterations or variants of unknown significance resulted in significantly higher clinical benefit rates (stable disease greater than or equal to 6 months, partial response, or complete response) than low alteration numbers in 69 patients evaluated with ctDNA next-generation sequencing testing. Gandera et al. evaluated blood based TMB in non-small cell lung cancer patients. Blood-based TMB correlated well with tumor-based TMB. Blood-based TMB could identify patients who would derive benefit from the anti-PDL1 antibody atezolizumab in second line therapy and beyond [39]. Georgiadis et al. saw improved outcomes for patients with metastatic cancer who were MSI or TMB-high on cfDNA analysis and treated with PD-1 inhibition [40]. Liquid biopsy-based TMB and MSI status thus have value in predicting response to immune checkpoint blockade, thus providing a valuable tool for predicting immune checkpoint blockade benefit.

### Predicting response and resistance early after targeted therapy and chemotherapy via serial liquid biopsies

Additional tools for early detection of response and resistance, prior to standard imaging studies, is important to help guide targeted therapy and chemotherapy treatment. Serial ctDNA and CTC evaluations have both been evaluated as tools for such outcome prediction.

Tie et al. evaluated 53 metastatic colorectal cancer patients receiving standard first-line chemotherapy with ctDNA measured before treatment and at two time points in the first cycle. Significant reductions in ctDNA were observed before cycle 2 and correlated with CT scan responses at 8–10 weeks [41]. Serial ctDNA testing during neoadjuvant treatment on the I-SPY2 TRIAL in 61 patients with positive ctDNA pretreatment found that those breast cancer patients who did not clear ctDNA were more likely to have residual disease. For patients who did not achieve a pathologic complete response, ctDNA positive patients were more likely to have metastatic recurrence [42].

Sirgavani et al. [43] studied ctDNA in patients with primary or acquired resistance to EGFR blockade. They identified alterations in *KRAS, NRAS, MET, ERBB2, FLT3, EGFR* and *MAP2K1* genes in ctDNA in patients with EGFR inhibitor resistance. Mutated *KRAS* clones, which emerge in blood during EGFR blockade, decline upon withdrawal of EGFR-specific antibodies, reflecting ongoing clonal evolution.

Cao et al. monitored ctDNA mutational changes during therapy in patients with advanced colorectal cancer receiving first line regimens and found that dynamic changes in ctDNA mutational status correlated with disease progression [44]. Ortiz-Cuaran et al. performed serial ctDNA evaluation in *BRAF*-mutated NSCLC patients receiving BRAF-directed therapies. A rebound in BRAF levels was observed in 60% of patients with progressive disease [45]. Razavi et al. evaluated ctDNA in hormone receptor-positive breast cancer patients on a phase I/II trial of the PI3K-alpha inhibitor alpelisib given with an aromatase inhibitor and found that loss of function PTEN mutations and ESR1-activating mutations emerged with resistance [46]. A study of serial ctDNA evaluations in HER2-positive metastatic breast cancer treated with oral anti-HER1/HER2 tyrosine kinase inhibitors by Ma et al. found that *HER2* amplification related to disease progression. The emergence or increase in the fraction of mutations of specific genes (*TP53/PIK3CA/MTOR/PTEN*) predicted resistance to therapy [47]. Dawson et al. evaluated the relationship between ctDNA, CA15-3, and imaging in metastatic breast cancer patients on systemic therapy. ctDNA had greater dynamic range and correlation with changes in tumor burden than CA15-3 and provided the earliest measure of response to treatment in 53% of women [48]. A retrospective study by Parkinson et al. evaluated high-grade serous ovarian cancer patients with serial ctDNA and found that *TP53* mutant allelic fraction decreases and pre-treatment levels were predictors of time to progression [49].

A phase II clinical trial of erlotinib and pertuzumab in advanced non-small cell lung cancer evaluated CTCs serially during treatment. Decreases in CTCs correlated well with radiographic response and improved progression-free survival [50].

Overall, serial evaluation of ctDNA and CTCs can provide early prediction of response and potential resistance to targeted therapy and chemotherapy, which can complement imaging studies and standard tumor markers.

### Assessing heterogeneity via ctDNA and CTCs, including single cell multi-omics

Metastatic solid tumors are heterogeneous, and sampling different sections of a tumor and different sites of disease may yield a distinct genomic profile. Providing a comprehensive molecular profile of the entire tumor is important for determining optimal therapeutic options for patients.

Single tumor-biopsy samples can lead to an underestimation of the tumor genomic landscape given intratumor heterogeneity [51]. However, ctDNA or CTCs may be shed into the blood from multiple metastatic sites and can be evaluated for a more complete picture. As an example, a prior study followed ctDNA and plasma samples in a patient with metastatic breast cancer to show that ctDNA can allow for real-time sampling of multifocal clonal tumor evolution [52].

Single-cell multi-omics is a new technology that can profile genomes, transcriptomes, proteomes, and epigenomes in single cells [53]. Application of single-cell multi-omics to CTCs has the potential to better describe tumor heterogeneity. For instance, therapy selected based on convergence pathways of molecular alterations may be ineffective if distinct molecular abnormalities occur in different cells; in such cases, co-targeting of each of the molecular abnormalities may be necessary. Thus, single-cell analysis of CTCs can allow for a better understanding of the molecular landscape of the entire cancer.

Current evidence suggests that ctDNA and CTCs can provide molecular information on the cancer as a whole; however, it is not clear if multiple metastatic lesions located in different organs shed ctDNA and CTCs homogenously [54]. ctDNA and CTCs may provide a more comprehensive view of the genomic landscape of the entire cancer and offer a safer and less expensive option than tissue biopsy of multiple tumor sites.

### Evaluating molecular alterations that are potentially actionable

Determining actionable alterations in metastatic solid tumors is critical for a precision medicine treatment approach. While this information was traditionally obtained from molecular profiling of tumor tissue biopsies, ctDNA/cfDNA and CTCs are proving valuable in this regard. There are, for instance, several blood-derived ctDNA‐based companion diagnostic tests approved by the Food and Drug Administration (FDA), e.g., the cobas epidermal growth factor receptor (EGFR) mutation test V2 to detect *EGFR* mutations in non‐small cell lung cancer and the therascreen *PIK3CA* RGQ PCR kit to detect *PIK3CA* mutations in breast cancer. The FDA has also approved Guardant360 liquid as well as FoundationOne liquid biopsy (both based on next-generation sequencing of ctDNA) as companion diagnostics for general molecular profiling and for several targeted therapies, with both potentially influencing patients’ therapy choices or making them eligible for clinical trials.

Additional ctDNA testing for druggable alterations has been studied. For instance, cancers of unknown primary (CUP) can present a challenge in determining therapy. These patients typically receive empiric chemotherapy with taxane- and/or platinum-based regimens with overall poor outcomes [55]. Kato et al. [56] evaluated 442 patients with CUP using next-generation sequencing of ctDNA. They found that 80% exhibited ctDNA alterations and 66% had pathogenic alterations. The most commonly altered genes were TP53-associated (38%), MAPK pathway (31%), and PI3K signaling genes (18%). Among 290 patients with pathogenic alterations, almost all (> 99%) had actionable alterations. A separate study [57] evaluated 1931 patients with CUP using cfDNA NGS panels; more than 90% had at least one ctDNA alteration. Level 1, 2 or resistance/R1 alterations were found in 47.4% of patients based on OncoKB classification. In a subset of patients who had clinically accessible data, those with higher degrees of matching between drugs given and molecular alterations had significantly improved clinical benefit rates (stable disease ≥ 6 months/partial response/complete response) compared to those with lower degrees of matching, suggesting clinical utility.

ctDNA/cfDNA can also help identify potentially actional mutations in other cancer types. Shatsky et al. studied 62 patients with advanced breast cancer and found that 68% had ≥ 1 pathogenic ctDNA alteration [58]. A study of 55 advanced, resected esophageal, gastroesophageal junction, and gastric adenocarcinoma reported that 69% of patients had ≥ 1 deleterious alteration [59]. In gynecologic cancers, Charo et al. demonstrated that therapy matched to ctDNA-identified alterations for 33 patients led to significantly improved survival [60]. A separate study showed *EGFR* amplifications in cfDNA in 8.5% of 28,584 pan-cancer patients. Responses were seen in 5 of 9 patients receiving EGFR inhibitors, including three patients who had amplifications in cfDNA, but not in tissue DNA [61]. In a cohort of colorectal cancer patients, Choi et al. assessed ctDNA and found at ≥ 1 pathogenic alteration in 76% of patients. All characterized alterations were potentially targetable with FDA-approved drugs or experimental drugs in clinical trials [62]. In a cohort of biliary tract cancers, Okamura et al. evaluated 121 patients receiving systemic treatment and found at least 76% of patients had ≥ 1 characterized alteration on ctDNA evaluation. Of these, 80 patients were treated, and those who had molecularly matched therapies based on genomic profiling by ctDNA and/or tissue DNA were found to have significantly longer progression-free survival and higher disease control rate than those on unmatched regimens [63]. Schwaederle et al. evaluated ctDNA in patients with non-small cell lung cancer and found that 82% had ≥ 1 one alteration potentially actionable by either an FDA-approved or an experimental clinical trial therapies [64]. Li et al. used ultra-deep plasma next-generation sequencing of cfDNA for non-small cell lung cancer and found a sensitivity of 75% with a specificity of 100% for identifying known targetable oncogenic driver mutations [65]. Maron et al. evaluated ctDNA in patients with gastroesophageal adenocarcinoma and was able to detect *HER2* and *EGFR* amplifications, which were predictive of benefit with HER2- and EGFR-directed therapy, respectively [66].

CTCs have been used to evaluate patients with metastatic, castration-resistant prostate cancer initiating taxane therapy. The detection of AR-V7 alterations in CTCs predicted greater efficacy of taxanes than androgen blockers, enzalutamide, or abiraterone therapy, whereas the absence of AR-V7 led to comparable efficacy [67].

Taken together, the literature suggests that the evaluation of molecular alterations found in ctDNA/cfDNA and CTCs has yielded actionable information in the majority of patients with cancers of unknown primary and a wide variety of other solid tumors. Therefore, liquid biopsy is a valuable tool that can be exploited along with molecular profiling of tissue biopsy to guide therapeutic decision making.

## Liquid biopsies as a prognostic tool including after surgery

Determining which patients are at risk for poor outcomes during or after treatment is critical for deciding on the appropriateness of more aggressive therapy and the frequency of monitoring. Both ctDNA/cfDNA and CTCs have been evaluated as prognostic tools.

Schwaederle et al. [68] evaluated ctDNA in a cohort of patients to determine if the % of ctDNA correlated with clinical outcomes. The median ctDNA variant allele fraction for each mutation was 0.45%. Patients who had at least one gene alteration with a ctDNA amount of greater than 5% had significantly worse progression-free and overall survival. A pan-cancer study by Vu et al. determined that the total number of alterations detected by ctDNA was independently associated with worse overall survival [69]. Jensen et al. [70] evaluated ctDNA with low-coverage genome-wide sequencing and found that the elevation of a calculated genomic instability number (GIN) correlated with worse survival. Baumgartner et al. evaluated pre-operative ctDNA in patients with peritoneal carcinomatosis and found that those with high levels of ctDNA had shorter progression-free survival after surgery independent of histologic grade [71].

A pan-cancer study comparing ctDNA and tissue DNA found that when *TP53* mutations were identical in tissue and ctDNA (16% of patients), survival was significantly shorter than for patients with differing mutations or no mutations detected [72]. A separate pan-cancer comparison of ctDNA and tissue found that the presence of *KRAS* alterations in both assays was an independent prognostic factor of poor survival [73]. Ikeda et al. evaluated *MET* alterations in a variety of tumor types by ctDNA and found that the presence of *MET* alterations correlated with bone metastases, *TP53* and *PTEN* alterations, and an increased number of overall alterations. *MET* alterations also correlated with a significantly shorter time to metastasis or recurrence along with worse overall survival [74].

In triple-negative breast cancer patients who were receiving or had completed neoadjuvant chemotherapy, the presence of ctDNA predicted significantly shorter disease-free survival. Pre-surgery, the detection of ctDNA predicted worse disease-free and overall survival [75]. In a cohort of locally advanced rectal cancer patients, the detection of post-operative ctDNA predicted recurrence regardless of the adjuvant chemotherapy administration and the presence of pathological complete response [76]. A study of pancreatic cancer patients by Patel et al. found that a higher total percentage of ctDNA was a prognostic factor for worse survival [77]. In patients with locally advanced squamous cell cancer of the anus, ctDNA detection after completion of chemoradiation was associated with a significantly worse disease-free survival [78]. A study of ctDNA by Anandappa et al. in stage II-III post-operative patients who had not started adjuvant chemotherapy found that patients with positive ctDNA had significantly higher rates of relapse [79]. Okamura et al. evaluated cfDNA in patients with invasive glioma following temozolomide and radiation. A significantly shorter overall survival was observed for patients with mutations that were due to clonal hematopoiesis [80]. Nie et al. evaluated blood-based TMB in non-small cell lung cancer. Blood TMB was negatively associated with clinical benefit of docetaxel [81].

A study of CTCs in metastatic breast cancer evaluated patients with measurable disease prior to starting treatment. A CTC number of greater than 5 per 7.5 ml of whole blood prior to treatment was an independent predictor of shorter progression-free and overall survival [82]. A separate multicenter study of metastatic breast cancer found an independent poor prognostic effect of CTC count of 5 per 7.5 mL or higher at baseline on progression-free and overall survival [83]. Non-metastatic breast cancer patients receiving adjuvant therapy with increases in CTC amount of tenfold or greater by the end of therapy had a strong likelihood of relapse [84]. The FDA has approved the CellSearch system for clinical use to detect CTCs in peripheral blood to predict outcomes for metastatic breast cancer patients.

Liquid biopsy has been extensively studied as prognostic tool and can provide valuable information on probable outcomes with therapy.

### Assessing difficult-to-biopsy patients

Molecular testing is limited for patients with tumors in hard-to-biopsy locations or who have co-morbidities reducing the ability to obtain tissue. Liquid biopsy may provide a methodology to obtain information to guide molecularly based therapeutic approaches in such patients. Jensen et al. [70] evaluated patients with a cancer diagnosis whose tumors were felt to be difficult to biopsy. cfDNA was isolated and low-coverage genome-wide sequencing was utilized successfully. This and other studies indicate that liquid biopsy can provide molecular information for patients in whom obtaining sufficient tissue to perform molecular profiling is difficult, dangerous, or not feasible.

### Early detection of cancer

In many malignancies, early detection and treatment of cancer is critical to improving outcomes. Identifying patients who have early-stage cancers amenable to surgery or radiation provides the best chance at long-term durable remissions. cfDNA, extracellular vesicles, and DNA methylation have been explored as methodologies for cancer screening.

A study of non-invasive prenatal testing (NIPT) utilized cfDNA to assess the fetal genome. Maternal and fetal contributions can often be co-mingled with this testing. An abnormal genomic profile that was not consistent with fetal abnormalities was identified in approximately 10 out of 100,000 cases. NIPT cases with these abnormalities were collected and catalogued with 55 total non-reportable altered maternal genomic profiles identified. Forty-three of the 55 cases had sufficient information to allow follow-up, and maternal neoplasm was eventually identified in 40 cases. These included 18 malignancies, 20 benign uterine fibroids, and 2 with radiological confirmation but without pathological classification. Thus, cfDNA may serve as an early biomarker for cancer [85].

Hinestrosa et al. [86] utilized biomarkers present in circulating extracellular vesicles in an attempt to detect early-stage, curable pancreatic, ovarian, and bladder cancers. Pathologically confirmed stage I and II cancer cases were compared to control subjects in a pilot study. Detection of stage I cancers were over 95% for pancreatic, 74% for ovarian, and ~ 44% for bladder cancer.

The CancerSEEK assay [87] utilized cfDNA to evaluate a large cohort of patients with nonmetastatic clinically detected cancers. Testing sensitivity ranged from 69 to 98% for cancers of the ovary, liver, stomach, pancreas, and esophagus, with a specificity of over 99%. These are cancers that do not have metastatic screening tests available; thus cfDNA may allow for screening and early detection.

Increases in DNA methylation of tumor suppressor genes are an early event in the development of many tumors. A hepatocellular-specific methylation marker was developed for diagnosis and monitoring of cancer [88]. This panel demonstrated superior sensitivity and specificity over AFP for hepatocellular carcinoma diagnosis. It also correlated well with tumor stage for early-stage tumors. Liu et al. explored targeted methylation sequencing in cfDNA in advanced colorectal cancer, non-small-cell lung cancer, breast cancer, and melanoma and found that methylation scores could accurately classify the presence of cancer in 83.8% of cases with 100% specificity. Methylation scores accurately predicted cancer type in 78.9% of cases [89]. A separate prospective study utilized targeted methylation analysis of cfDNA in over 6600 cases and was found to have a specificity of 99% and a sensitivity of 67% across 12 common cancer types (44% for all cancer types) in identifying stage I-III disease [90].

Thus, blood-based cfDNA, extracellular vesicles, and DNA methylation have all been explored as cancer screening methodologies. Liquid biopsy has the potential to accurately detect early-stage cancer, which may allow for timely intervention, and has the potential to prevent the development of advanced disease.

### Predicting CAR-T cell response

CAR-T cells are increasingly used for treatment of hematologic malignancies, with remarkable responses. However, additional tools to predict response and monitor for progression beyond imaging and bone marrow biopsy are important in order to further improve outcomes. A pilot study of 12 patients used the blood-derived GIN analysis to evaluate response to CAR-T cell therapy for patients with aggressive B-cell lymphomas [91]. cfDNA was isolated prior to treatment and sequentially after CAR-T cell infusion. All five patients who remained in complete response throughout the study had GIN less than the threshold of 170. In five of six patients with relapsed or progressive disease, increasing GIN was observed before imaging diagnosis. Therefore, cfDNA has the potential to allow early prediction of relapse or progressive disease and can be serially collected to monitor response after CAR T-cell infusion. Additional studies in this area are needed.

### cfDNA fragmentation

A newer application is the evaluation of cfDNA fragmentation. cfDNA fragmentations non-randomly occur in blood circulation and often depend on the tissue of origin. The cfDNA fragmentome can comprehensively represent both genomic and chromatin characteristics. Mathios et al.[92] used cfDNA fragmentation to evaluate 365 individuals at risk for lung cancer and found that a model combining fragmentation features, clinical risk factors, and CEA levels followed by CT imaging was able to detect 94% of patients with lung cancer across stages and subtypes. The fragmentation profiles were also able to distinguish individuals with small cell lung cancer from those with non-small cell lung cancer with high accuracy. Cristiano et al. [93] evaluated fragmentation profiles across 236 patients with multiple cancer types and compared to healthy individuals with a machine learning model and found that these profiles could be used to identify the tissue of origin of cancers with 61% accuracy and increased to 75% when assigning cfDNA to one of two sites of origin. Jiang et al. [94] evaluated DNA end characteristics in patients with hepatocellular carcinoma and chronic hepatitis B. They found that cell-free DNA fragments with ends at certain genomic coordinates had higher probability of coming from hepatocellular carcinoma. Guo et al. [95] evaluated cfDNA fragmentomes in 292 stage I invasive lung adenocarcinomas along with healthy volunteers and were able to develop a model that was more than 92% sensitive for early-stage minimally invasive and small (< 1 cm) tumors. Thus, cfDNA fragmentation analysis has the potential to help identify cancers and even cancer type.

### Alternate fluids

While ctDNA/cfDNA have traditionally been procured from the blood, isolation from other fluids has also been explored, albeit to a lesser extent. Accessing ascites, pleural fluid, urine and cerebrospinal fluid (CSF), may provide complementary information to blood or tissue findings, and serial urine samples are simple to obtain.

Husain et al. evaluated *EGFR*-activating and EGFR-resistance mutation levels in daily urine samples of patients with non-small cell lung cancer receiving the EGFR inhibitor osimertinib. Radiographic responses seen at weeks 6 to 12 were preceded by a pattern of a large spike of *EGFR* ctDNA early in week 1, followed by a dramatic decline, usually by day 5, suggesting apoptosis with early release of ctDNA [96]. Tong et al. evaluated cfDNA from pleural fluid and found higher levels of tumor DNA than plasma samples. They found that 93% of tissue-derived tumor driver mutations including *ALK, BRAF, EGFR, ERBB2, KRAS, NF1, PIK3CA*, and *RET* were found in pleural cfDNA as compared to only 62% in plasma-derived cfDNA [97]. Han et al. explored ctDNA from ascites fluid in patients with epithelial ovarian cancer. Of 10 patients evaluated, 9 had somatic mutations in both tumor and ascites ctDNA, with the most common mutated gene being *TP53*; thus ascites ctDNA was felt to be helpful in identifying the mutational landscape of ovarian cancer [98]. Bobillo et all explored ctDNA levels in CSF of lymphoma patients. ctDNA was found in the CSF of all patients with CNS restricted lymphomas, but not for systemic lymphomas without CNS involvement. ctDNA also predicted CNS relapse [99].

Currently, most liquid biopsies are derived from blood samples. However, urine samples are advantageous as multiple urine “biopsies” can be obtained per day. On the other hand, urine ctDNA may be more fragmented, and larger segments difficult to analyze [100]. We have just touched the tip of the iceberg with additional fluids such as effusions, ascites and CSF, which conceivably could reveal underlying heterogeneity and causal alterations leading to fluid accumulation or, in the case of brain or leptomeningeal disease, important underlying drivers not easily shed into the blood.

### Advantages and limitations of liquid biopsies

**Fig. 2:**
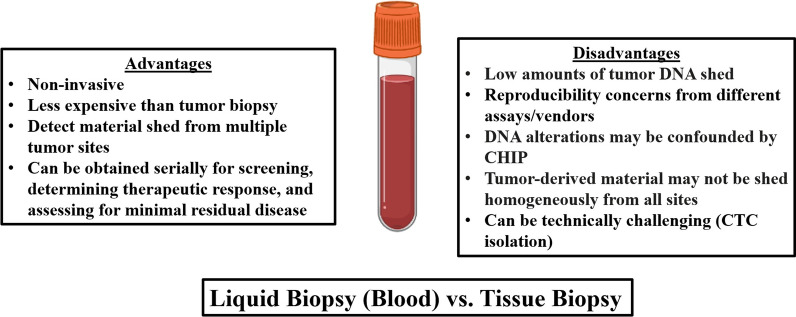
Advantages and disadvantages of liquid versus tissue biopsy. Liquid biopsies are non-invasive, less expensive, can assess multiple tumor sites, and can be obtained serially. Low tumor DNA shed, CHIP, and reproducibility issues may limit usage. CTC isolation can also be technically challenging. Image created with help of Biorender.com. CHIP: clonal hematopoiesis of indeterminate potential, CTCs: circulating tumor cells

Liquid biopsies have many advantages (Fig. 2). They are non-invasive and less expensive than traditional tissue biopsies. They have the potential to detect material shed from multiple metastatic sites rather than analyzing a small piece of tissue biopsied; therefore, liquid biopsies have the potential to better detect heterogeneity in the tumor across sites. Liquid biopsies can be obtained serially to observe changes with therapy. They are an easier means for monitoring therapeutic responses than tissue biopsy, and liquid biopsies have potential in early cancer detection as part of screening, as well as detecting minimal residual disease following therapy.

There are also several limitations of liquid biopsies (Fig. 2). ctDNA/cfDNA can be shed in only small amounts and not all patients will have detectable levels, especially those with low tumor burden. Because of the small amount of material shed in the circulation, sequencing can be difficult and expensive. Standardization across laboratories and vendors is needed to ensure reproducibility. Not all detectable cfDNA alterations are cancer-related; indeed, cfDNA may be confounded by the mutations derived clonal hematopoiesis of indeterminate potential (CHIP), especially in older patients [80]. Moreover, not all ctDNA/cfDNA is equally shed from the primary tumor and metastases, so it is unclear if the alterations detected accurately represent tumor heterogeneity. Shedding of ctDNA can be suppressed by treatment and may be limited at certain disease sites [1].

The isolation of CTCs remains technologically challenging, and the number of CTCs isolated can be method dependent. Surface markers may be downregulated in certain tumors, which can limit the ability to detect CTCs [101]. It is unclear if the CTCs are uniformly shed from all areas of the primary tumor and metastases; thus, the CTCs isolated may not provide a full portfolio of tumor heterogeneity [102]. On the other hand, CTCs can provide DNA, RNA and protein results and can be cultured for functional studies.

## Conclusions

Liquid biopsies have emerged as a remarkable technology, with clinical applications almost unimaginable a decade ago. They are increasingly being used for molecular profiling of tumors and for facilitating a precision medicine treatment approach.

Tumors release ctDNA/cfDNA and CTCs into the blood stream. A number of technologies are used to isolate and analyze ctDNA/cfDNA including, but not limited to, ddPCR, BEAMing, TAm-Seq, CAPP-Seq, WGBS-Seq, whole exome sequencing, and whole genome sequencing. Isolation of CTCs can be performed by a variety of methods including capturing cells using specific biomarkers expressed on the cell surface, and a range of enrichment techniques based on biophysical properties.

Both ctDNA/cfDNA and CTCs can be exploited for multiple applications. These include, but are not limited to, differentiating unique immune checkpoint blockade response patterns, predicting immune checkpoint blockade and CAR T-cell response, assessing shed DNA from multiple metastatic sites in order to understand tumor heterogeneity, assessing pharmacologically tractable alterations, analyzing serial levels for response prediction and early detection of resistance after chemotherapy and targeted therapy or for assessing residual disease, as a prognostic tool in multiple settings including pre- and post-operatively, assessing difficult-to biopsy patients, and detection of cancer at its earliest stages. While ctDNA/cfDNA and CTCs are generally isolated from the blood, other fluids including urine, CSF, ascites, and pleural fluid are being explored. ctDNA/cfDNA is easier to isolate than CTCs, but can be limited by small amounts in the bloodstream and confounded by CHIP. CTCs have an advantage in that their use can allow for protein, DNA, and RNA evaluation, including at the single-cell level. Furthermore, CTCs can be cultured or developed in xenografts, which may enable functional appraisal of response and resistance to therapies prior to patient administration.

ctDNA/cfDNA and CTCs are both easier to collect serially (and less expensive) than tissue biopsy and, in many cases, can provide critical molecular and response information in real time, especially for patients harboring difficult-to-biopsy neoplasms. These novel liquid technologies can be utilized along with tissue DNA to determine molecular-guided therapy. They can also be used in conjunction with imaging to provide valuable information regarding clinical response, resistance, and prognosis. There are already several FDA-approved liquid biopsy companion diagnostics, such as those for detecting *EGFR* or *PIK3CA* alterations, both of which are druggable. The FDA has also approved both FoundationOne Liquid CDx and Guardant360 liquid CDx as companion diagnostics for several targeted therapies as well as use of the tests for general tumor profiling, which may influence patients’ treatment choices or make them eligible for clinical trials.

There are also certain limitations to liquid biopsies and challenges in this field. The Accelerating Anticancer Agent Development Workshop expert panel outlined these issues in a session entitled “Liquid Biopsy: State of the Science and Future Directions.” The panel identified challenges such as standardization of liquid biopsy assessments and analyte validation, as well as regulatory considerations for their use as a biomarker in clinical trials [103].

There are multiple potential future directions that are emerging for liquid biopsies. They include use of alternate fluids (CSF, ascites, effusions, urine, etc.) and exploration of additional analytes such as circulating tumor RNA, cell‐free micro-RNA, and exosomes. Early detection of cancer at a point where it is curable is also an area of vigorous, and potentially transformative, research. Thus, liquid biopsy is a powerful, multifaceted tool to help improve oncology management and outcomes.

## Data Availability

Not applicable (review article).

## Abbreviations

BEAMing: Beads, emulsion, amplification, and magnetics
CAPP-Seq: Cancer personalized profiling by deep sequencing
CHIP: Clonal hematopoiesis of indeterminate potential
ddPCR: Droplet digital polymerase chain reaction
cfDNA: Cell-free DNA
CSF: Cerebrospinal fluid
CTC: Circulating tumor cells
ctDNA: Circulating tumor DNA
EpCAM: Epithelial cell adhesion molecule
GIN: Genome instability number
TAm-Seq: Tagged-amplicon deep sequencing
WES: Whole exome sequencing
WGS: Whole genome sequencing
WGBS-Seq: Whole genome bisulfite sequencing
